# Representation of Cancer in the Medical Literature - A Bibliometric Analysis

**DOI:** 10.1371/journal.pone.0013902

**Published:** 2010-11-09

**Authors:** Ronan W. Glynn, Ji Z. Chin, Michael J. Kerin, Karl J. Sweeney

**Affiliations:** Department of Surgery, Clinical Science Institute, National University of Ireland Galway, Galway, Ireland; The Wistar Institute, United States of America

## Abstract

**Background:**

There exists a lack of knowledge regarding the quantity and quality of scientific yield in relation to individual cancer types. We aimed to measure the proportion, quality and relevance of oncology-related articles, and to relate this output to their associated disease burden. By incorporating the impact factor(IF) and Eigenfactor™(EF) into our analysis we also assessed the relationship between these indices and the output under study.

**Methods:**

All publications in 2007 were retrieved for the 26 most common cancers. The top 20 journals ranked by IF and EF in general medicine and oncology, and the presence of each malignancy within these titles was analysed. Journals publishing most prolifically on each cancer were identified and their impact assessed.

**Principal Findings:**

63260 (PubMed) and 126845 (WoS) entries were generated, respectively. 26 neoplasms accounted for 25% of total output from the top medical publications. 5 cancers dominated the first quartile of output in the top oncology journals; breast, prostate, lung, and intestinal cancer, and leukaemia. Journals associated with these cancers were associated with much higher IFs and EFs than those journals associated with the other cancer types under study, although these measures were not equivalent across all sub-specialties. In addition, yield on each cancer was related to its disease burden as measured by its incidence and prevalence.

**Conclusions:**

Oncology enjoys disproportionate representation in the more prestigious medical journals. 5 cancers dominate yield, although this attention is justified given their associated disease burden. The commonly used IF and the recently introduced EF do not correlate in the assessment of the preeminent oncology journals, nor at the level of individual malignancies; there is a need to delineate between proxy measures of quality and the relevance of output when assessing its merit. These results raise significant questions regarding the best method of assessment of research and scientific output in the field of oncology.

## Introduction

Proportional representation in the medical literature of individual cancers and oncology as a whole has been difficult to establish. The rapid increase in medical research publications has been facilitated by the development of the internet, integrated search engines, and on-line publishing. The two principal repositories for medical research publications are the Web of Science (WoS) (Thompson Reuters), and PubMed (the National Library of Medicine (NLM)), the latter recognised as the most frequently used source for information in the medical field [1]. Recently developed internet-based analytical tools now allow for interrogation of these online databases and for the provision of reports that are comparable within and between datasets.

Bibliometrics is a systematic method for evaluating research output which can help map changes in the interest of a scientific community over time, [2] and can provide insights into both qualitative and quantitative research trends. The bibliometric indicator most commonly used to undertake qualitative analysis is the journal impact factor (IF) which is based on two elements; the numerator, which is the number of citations in the current year to items published in the previous 2 years, and the denominator, which is the number of substantive articles and reviews published in the same 2 years [3]. Despite its popularity, there are a number of criticisms of the IF, particularly surrounding the ease with which it may be manipulated by journals [4], and the lack of clarity regarding what constitutes a ‘citable’ output [5]. It has further been argued that the metric lacks normalisation for reference practices across disciplines [6], [7], and does not indicate the relevance (the degree to which a journal publishes on a particular topic or sub-specialty) of publications to a particular audience. In contrast, the recently developed Eigenfactor™ (EF), which ranks journals according to the number and weight of incoming citations, attempts to adjust for differences in “citation culture” between journals and across fields, and may provide an enhanced level of discrimination [8].

Calculation of the EF is based on that which Google uses when ranking web pages. This PageRank algorithm [9] regards the hyperlinks (links on web pages which connect users to other web pages) as recommendations, with two extra comments: (1) the status of the recommender is important, and (2) the recommendation should drop in weight if the recommender is too generous giving them. In short, a web page is important (gets a high popularity score) if it is pointed to by other important (high ranked) pages [10]. Instead of websites, the Eigenfactor algorithm scores journals, and instead of using hyperlinks, it uses citations. By simulating random traffic on a network these algorithms calculate the popularity of journals in a self-consistent fashion. Its developers claim that the resulting score provides an estimate of the percentage of time that users spend with a particular journal, with this amount of time postulated to be a measure of that journal's influence within the overall network of academic citations [11]. The resulting rankings for journals have been published on Eigenfactor.org since 2006 [11], and are now published as part Thomson Reuter's annual Journal Citation Report (JCR).

Bibliometric analysis has previously been employed as a method of correlating research productivity in oncology with geographic variation in output and funding [12], [13], and the development of translational research [14]. Investigation of output across a range of disciplines within oncology has not been undertaken previously however, nor has an attempt been made to relate this output to proxy measures of quality such as the IF and EF. The principal objective of this study therefore, was to measure the proportion, quality and relevance of articles for the most common cancer types. By incorporating the IF and EF into our analysis we also aimed to assess the relationship between these bibliometric indices and the research output under study.

## Materials and Methods

Publications were retrieved by searching for each cancer using its medical subject heading (MeSH) term in PubMed. The subheadings encompassed by each MeSH term were then employed to perform an equivalent search in the WoS database. Numbers were obtained for English-language entries for each of the malignancies under study. All peer-reviewed articles, including editorials, reviews, technical notes and letters to the editors were included.

Both PubMed and the WoS databases were consulted for the reference period 01/01/2007 to 31/12/2007, with all searches conducted between May and August 2009. Search results in the WoS included entries from the “Science Citation Index-Expanded” and the “Social Sciences Citation” indices, yielding 126845 articles. Search results for PubMed, which covers Medline and other specialised databases within the National Library of Medicine (NLM), and catalogues entries from 6000 journals, yielded 63260 articles.

The 26 cancers with the highest incidence as defined by the Surveillance, Epidemiology, and End Results (SEER) database of the National Cancer Institute (NCI) in 2006 were included in the study (Table S1) [15]. The cancer with the 27th highest incidence in this database was that involving bone; this was not included due to the potential confounding influence of publications relating to bone metastases on our analysis.

Three collections of journals were included in this analysis.

Cluster A (Table S2):

AIF-The top 20 medical journals ranked by impact factor, 2007

AEF-The top 20 medical journals ranked by eigenfactor, 2007

Cluster B (Table S2):

BIF-The top 20 oncology journals ranked by impact factor, 2007

BEF-The top 20 oncology journals ranked by eigenfactor, 2007

Cluster C (Table S3):

The ten journals which published most prolifically on each of the 26 cancers.

Clusters A and B were identified in the 2007 edition of Thomson Reuter's JCR, and cluster C was identified using the cloud-based web service PubReminer [16].

In order to assess scientific yield relative to disease burden, a publication ratio was derived using a method described by Al Shahi et al. in 2001 [17]. Briefly, we divided the number of Pubmed papers published in 2007 about each cancer by a measure of its disease burden (incidence or prevalence). Incidence and prevalence data were obtained from the SEER database [15].

Each relevant bibliographic record was downloaded and then evaluated and assessed using Microsoft Excel spreadsheet software, and Statistical Package for Social Science version 15.0 (SPSS Inc, Chicago, Illinois, USA) software. The relationship between IF and EF values was investigated using the Spearman correlation coefficient and Kruskal-Wallis between groups analysis; a p value of less than 0.05 was deemed statistically significant.

## Results

Publications on the 26 cancers under study accounted for 8.19% (63260/772243) of output in PubMed, and 8.04% of output in WoS (126845/1576018). Breast cancer accounted for the highest percentage of oncology publications in both Pubmed (13·81%) and the WoS (13·83%) (Table 1). Other high publication rate cancer subjects (present in the top quartile of output for both databases) included lung cancer, leukaemia, intestinal cancer, and prostatic cancer.

**Table 1:** Number (Percentage) of Publications For Neoplasms In PubMed and the Web of Science (WoS).

PubMed				WoS		
Quartile	Rank	Cancer	Output (%)	Rank	Cancer	Output (%)
1	1	Breast	8736 (13.81)	1	Breast	17563 (13.83)
1	2	Intestinal	6304 (9.97)	2	Intestinal	11305 (8.91)
1	3	Lung	5127 (8.10)	3	Lung	11237 (8.86)
1	4	Leukemia	4762 (7.53)	4	Leukemia	10226 (8.06)
1	5	Prostatic	4606 (7.28)	5	Pancreatic	9118 (7.19)
1	6	CNS	4106 (6.49)	6	Prostatic	8573 (6.76)
2	7	Liver	3877 (6.13)	7	Liver	7091 (5.59)
2	8	Uterine	3181 (5.03)	8	CNS	6664 (5.25)
2	9	Lymphoma	2801 (4.43)	9	Lymphoma	5270 (4.15)
2	10	Melanoma	2371 (3.75)	10	Kidney	4563 (3.60)
2	11	Ovarian	2235 (3.53)	11	Melanoma	4398 (3.47)
2	12	Stomach	1938 (3.06)	12	Ovarian	4153 (3.27)
2	13	Pancreatic	1937 (3.06)	13	Soft Tissue	3826 (3.02)
2	14	Kidney	1788 (2.83)	14	Mouth	3348 (2.64)
3	15	Mouth	1545 (2.44)	15	Stomach	3143 (2.48)
3	16	Urinary	1208 (1.91)	16	Hodgkin	3130 (2.47)
3	17	Esophageal	1191 (1.88)	17	Myeloma	2683 (2.12)
3	18	Thyroid	1182 (1.87)	18	Uterine	2378 (1.87)
3	19	Soft Tissue	1112 (1.76)	19	Urinary	2259 (1.78)
3	20	Myeloma	1085 (1.72)	20	Thyroid	1991 (1.57)
4	21	Testicular	568 (0.90)	21	Esophageal	1454 (1.15)
4	22	Hodgkin	500 (0.79)	22	Testicular	1017 (0.80)
4	23	Laryngeal	442 (0.70)	23	Mesothelioma	567 (0.45)
4	24	Mesothelioma	336 (0.53)	24	Laryngeal	526 (0.41)
4	25	Gallbladder	163 (0.26)	25	Gallbladder	213 (0.17)
4	26	Vulvar	159 (0.25)	26	Vulvar	149 (0.12)
		**Total**	63260 (100)		**Total**	126845 (100)

Neoplasms are listed in order of their decreasing contribution to the overall research output for the 26 neoplasms in each database, in 2007.

Oncology related articles constituted 25% of all publications in the top 20 medical journals according to IF (AIF, Oncology articles/total articles: 3096/12399) and EF (AEF, Oncology articles/total articles: 4689/18431) (Table 2). Almost two thirds of these articles related to just 6 cancers: prostate, breast, intestinal, lung, non-hodgkin lymphoma, and leukaemia (AIF, 65.5%, 2027/3096; AEF, 63.6%, 2982/4689).

**Table 2:** Number (Percentage) of Publications in the top 20 medical journals ranked by IF and EF for 26 most common neoplasms.

AIF			AEF		
Rank	Cancer	Output (%)	Rank	Cancer	Output (%)
1	Leukemia	533 (17.22)	1	Breast	732 (15.61)
2	Breast	496 (16.02)	2	Leukemia	646 (13.78)
3	Intestinal	348 (11.24)	3	Lung	493 (10.51)
4	Lung	266 (8.59)	4	Intestinal	482 (10.28)
5	Non-Hodgkin	214 (6.91)	5	Prostatic	353 (7.53)
6	Prostatic	170 (5.49)	6	Non-Hodgkin	276 (5.89)
7	Liver	162 (5.23)	7	CNS	208 (4.44)
8	Myeloma	123 (3.97)	8	Ovarian	205 (4.37)
9	CNS	105 (3.39)	9	Melanoma	194 (4.14)
10	Ovarian	86 (2.78)	10	Liver	165 (3.52)
11	Pancreatic	86 (2.78)	11	Myeloma	156 (3.33)
12	Stomach	81 (2.62)	12	Pancreatic	151 (3.22)
13	Uterine	73 (2.36)	13	Kidney	112 (2.39)
14	Kidney	61 (1.97)	14	Uterine	109 (2.32)
15	Hodgkin	60 (1.94)	15	Stomach	80 (1.71)
16	Melanoma	60 (1.94)	16	Hodgkin	65 (1.39)
17	Esophageal	48 (1.55)	17	Esophageal	54 (1.15)
18	Testicular	38 (1.23)	18	Urinary	48 (1.02)
19	Soft Tissue	29 (0.94)	19	Testicular	36 (0.77)
20	Urinary	21 (0.68)	20	Thyroid	35 (0.75)
21	Mesothelioma	11 (0.36)	21	Soft Tissue	30 (0.64)
22	Mouth	11 (0.36)	22	Mouth	26 (0.55)
23	Thyroid	9 (0.29)	23	Mesothelioma	24 (0.51)
24	Laryngeal	5 (0.16)	24	Laryngeal	6 (0.13)
25	Gallbladder	0 (0.00)	25	Gallbladder	2 (0.04)
26	Vulvar	0 (0.00)	26	Vulvar	1 (0.02)
	**Total**	3096 (100)		**Total**	4689 (100)

AIF, output on each malignancy in the top twenty medical journals by IF in 2007; AEF, output on each malignancy in the top twenty medical journals by EF in 2007.

Articles relating to the 26 cancers with the highest incidence constituted 53% and 72% of all publications in the top 20 oncology journals according to IF (BIF, 5086/9527) and EF (BEF, 8775/12209), respectively (Table 3). Two thirds of these articles were related to just 7 cancers: breast, prostate, lung, intestinal cancer, leukaemia, ovarian cancer and cancers involving the CNS (BIF, 63.5%, 3230/5086; BEF, 65.5%, 5746/8775).

**Table 3:** Number (Percentage) of Publications in the top 20 oncology journals ranked by IF and EF for 26 most common neoplasms.

BIF			BEF		
Rank	Cancer	Output (%)	Rank	Cancer	Output (%)
1	Breast	833 (16.38)	1	Breast	1595 (18.18)
2	Leukemia	638 (12.54)	2	Intestinal	895 (10.20)
3	Intestinal	511 (10.05)	3	Lung	862 (9.82)
4	Lung	497 (9.77)	4	Prostatic	851 (9.69)
5	Prostatic	489 (9.61)	5	Leukemia	759 (8.64)
6	CNS	262 (5.15)	6	Ovarian	398 (4.54)
7	Non-Hodgkin	246 (4.84)	7	CNS	386 (4.40)
8	Ovarian	239 (4.70)	8	Non-Hodgkin	373 (4.25)
9	Melanoma	199 (3.91)	9	Uterine	339 (3.86)
10	Liver	174 (3.42)	10	Liver	333 (3.79)
11	Pancreatic	160 (3.15)	11	Melanoma	321 (3.66)
12	Kidney	140 (2.75)	12	Pancreatic	278 (3.17)
13	Myeloma	128 (2.52)	13	Stomach	225 (2.56)
14	Uterine	119 (2.34)	14	Kidney	217 (2.47)
15	Stomach	91 (1.79)	15	Myeloma	166 (1.89)
16	Esophageal	71 (1.40)	16	Esophageal	160 (1.82)
17	Urinary	66 (1.30)	17	Urinary	141 (1.60)
18	Thyroid	45 (0.88)	18	Mouth	98 (1.11)
19	Hodgkin	44 (0.87)	19	Hodgkin	89 (1.01)
20	Testicular	40 (0.79)	20	Testicular	74 (0.84)
21	Mouth	32 (0.63)	21	Thyroid	73 (0.83)
22	Mesothelioma	27 (0.53)	22	Mesothelioma	51 (0.58)
23	Soft Tissue	23 (0.45)	23	Soft Tissue	42 (0.48)
24	Laryngeal	9 (0.18)	24	Laryngeal	36 (0.41)
25	Gallbladder	2 (0.04)	25	Gallbladder	9 (0.10)
26	Vulvar	1 (0.02)	26	Vulvar	4 (0.05)
	**Total**	5086		**Total**	8775

BIF, output on each malignancy in the top twenty oncology journals by IF in 2007; BEF, output on each malignancy in the top twenty oncology journals by EF in 2007.

**Figure 1:**
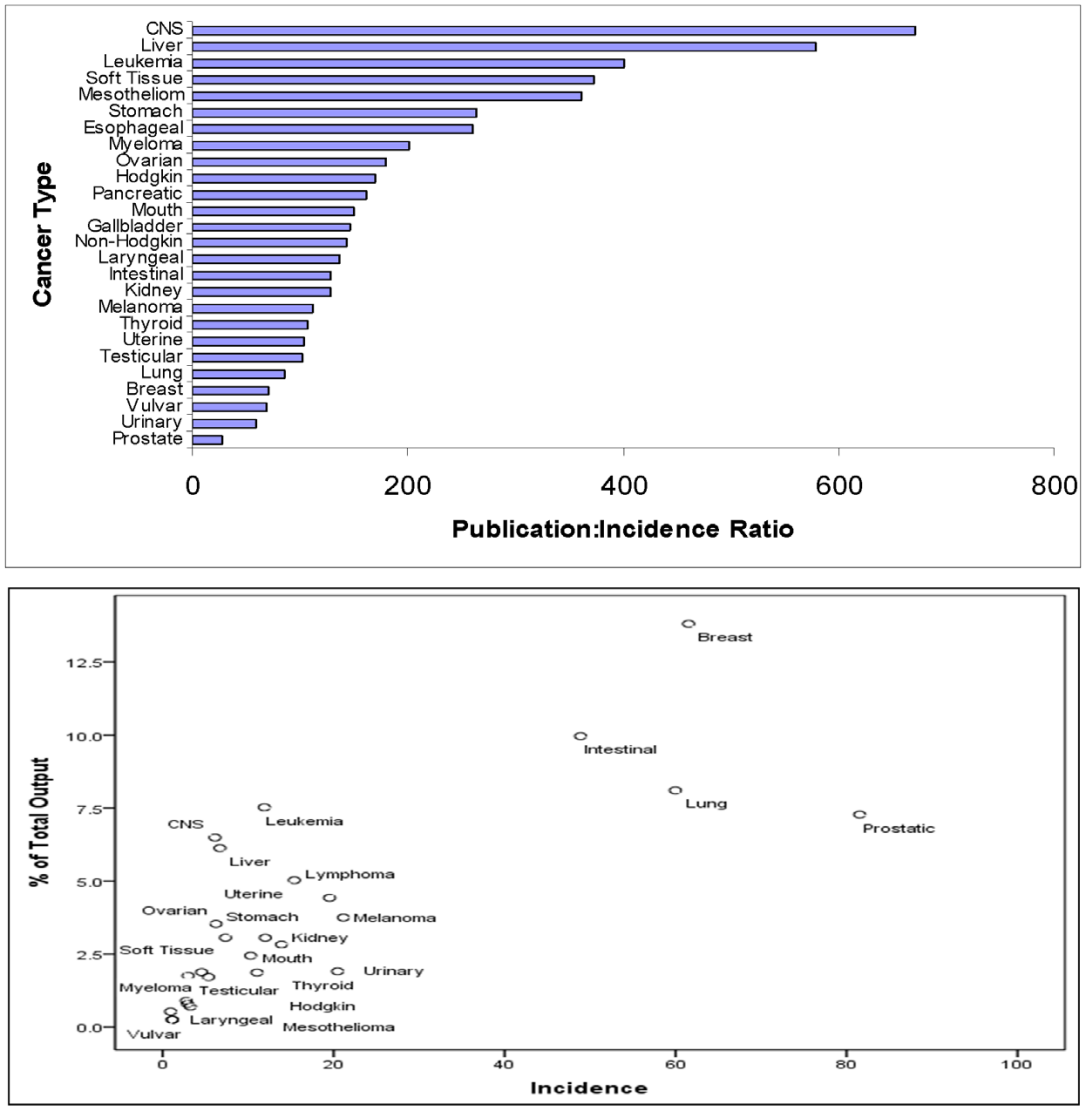
Number and Percentage of Publications, Relative to Cancer Incidence. (a) Publication ratios for 26 most common cancers, ordered by their incidence (b) Percentage of Total Publications Versus Cancer Incidence.

**Figure 2:**
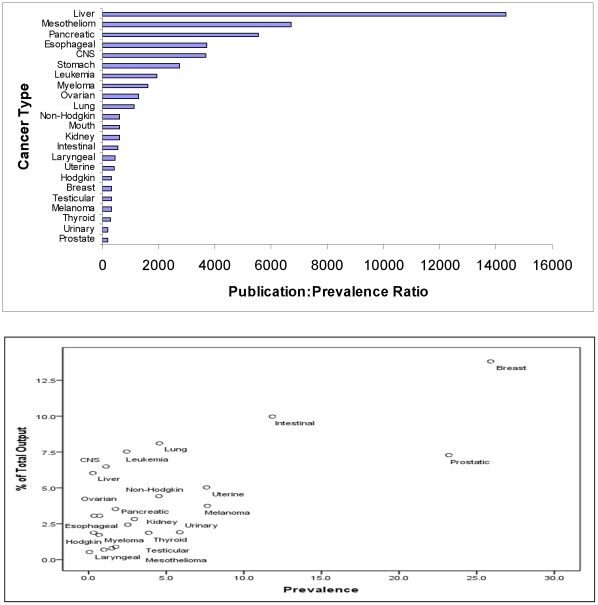
Number and Percentage of Publications, Relative to Cancer Prevalence. (a) Publication ratios for 23 most common cancers, ordered by their prevalence. Prevalence data was not available for 3 of the cancers; cancer of the gallbladder, vulva, and those involving the soft tissue and heart, (b) Percentage of Total Publications Versus Cancer Prevalence.

Figure 1(a) demonstrates the results of running our publication : incidence ratio. When research output is related to the actual incidence of each cancer, leukaemia and cancers involving the liver and central nervous system (CNS) appear overrepresented. The publication ratio for the latter, for example, was approximately 23-fold greater than that for prostate cancer and almost 10-fold greater than that for breast cancer. This is again highlighted in Figure 1(b), where cancer incidence is plotted against the percentage contribution to the overall research yield contributed by each cancer in 2007. Output was next assessed relative to associated disease prevalence. Cancers involving the liver and pancreas were markedly overrepresented (Figure 2(a)), with the publication ratio for the former more than 70-fold and 40-fold greater than that for prostate or breast cancer, respectively; similarly, when each cancer's percentage contribution to the total output is plotted against associated disease prevalence, it is evident that liver cancer and cancers involving the CNS are associated with high levels of research yield (Figure 2(b)).

The top 5 oncology journals by IF and by EF were in the top 10 most frequently publishing journals for 11 and 18 of the malignancies under study, respectively (Table S3). The journals which published on the widest range of cancers under study were the *Journal of Clinical Oncology* (n = 11), the *Annals of Surgical Oncology* (n = 11), and *Clinical Cancer Research* (n = 9).

The ten journals which published most prolifically on each cancer (cluster ‘C’) generated 16219 articles on the 26 cancers of interest in 2007 (Table S3). Over half of these articles were related to just 6 cancer sites: breast, leukaemia, prostate, lung, intestinal cancer, and cancers involving the CNS (n = 8570; 52%) (Table 4). There was no correlation between the total output of these journals and either the IF (p = 0.073) or the EF (p = 0.053). Hodgkin lymphoma, melanoma, lung and prostate cancer were located in the top quartile by both IF and EF, whilst cancers of the gallbladder, vulva, larynx and mouth were located in the lowest quartile by both measures. Significant differences were noted in the IFs and EFs of those cancers located in the upper quartile versus those in the lowest (median IF upper quartile 4.45 (IQR 4.20–4.61) versus median IF bottom quartile 1.66 (IQR 1.37–2.13), p = 0.000; median EF upper quartile 0.0515 (IQR 0.0451–0.0715) versus median EF lower quartile 0.0136 (IQR 0.0108–0.0187), p = 0.000).

**Table 4:** Number of Publications, median IF and median EF, of the top ten publishing journals on each malignancy.

Output			IF		EF	
Cancer	Output	Rank	IFMedian (IQR)	Rank	EFMedian (IQR)	Rank
Breast	1890	1	4.50 (3.47–3.57)	3	.0407 (0.0157–0.1707)	9
Leukaemia	1757	2	3.75 (2.01–7.92)	10	.0254 (0.0132–0.1238)	18
Prostatic	1505	3	4.17 (2.60–5.79)	6	.0643 (0.0272–0.1410)	2
Lung	1273	4	4.22 (2.02–6.61)	5	.0931 (0.0286–0.2324)	1
Intestinal	1144	5	4.04 (2.08–5.29)	7	.0374 (0.0171–0.1707)	13
CNS	1001	6	1.92 (1.08–3.33)	23	.0385 (0.0082–0.0525)	12
Uterine	895	7	2.77 (1.35–4.36)	17	.0424 (0.0104–0.0874)	8
Ovarian	708	8	3.62 (1.24–6.61)	12	.0440 (0.0038–0.2324)	7
Liver	690	9	2.42 (1.97–6.90)	20	.0182 (0.0116–0.0660)	22
Non Hodgkin	676	10	4.68 (2.30–7.92)	1	.0396 (0.0127–0.1238)	10
Kidney	517	11	3.40 (1.54–5.79)	14	.0451 (0.0090–0.1310)	5
Mouth	495	12	1.40 (1.13–1.63)	25	.0109 (0.0051–0.0189)	25
Urinary	438	13	2.66 (1.79–4.57)	18	.0340 (0.0053–0.1104)	15
Melanoma	437	14	4.41 (2.69–6.29)	4	.0487 (0.0116–0.2188)	4
Stomach	437	15	3.17 (1.50–4.36)	16	.0203 (0.0172–0.0758)	21
Pancreatic	431	16	3.92 (2.18–6.07)	8	.0388 (0.0136–0.1611)	11
Myeloma	388	17	3.75 (2.30–6.42)	9	.0254 (0.0127–0.0937)	18
Thyroid	306	18	3.30 (2.93–4.37)	15	.0231 (0.0140–0.0271)	20
Esophageal	271	19	2.30 (1.55–5.94)	21	.0347 (0.0200–0.0692)	14
Laryngeal	203	20	1.29 (0.63–1.85)	26	.0117 (0.0058–0.0218)	24
Soft tissue	190	21	2.47 (1.87–3.98)	19	.0279 (0.0132–0.0590)	16
Testicular	176	22	3.55 (1.95–8.09)	13	.0451 (0.0061–0.1612)	5
Hodgkin	162	23	4.59 (2.63–6.86)	2	.0544 (0.0213–0.1712)	3
Mesothelioma	88	24	3.69 (1.83–5.03)	11	.0266 (0.0049–0.0876)	17
Vulvar	84	25	1.43 (0.98–2.08)	24	.0106 (0.0050–0.0179)	26
Gallbladder	57	26	2.07 (1.57–2.35)	22	.0155 (0.0112–0.0224)	23

By EF, titles publishing most frequently on testicular and kidney neoplasms were joint 5th, and leukaemia and myeloma joint 18th. IQR  =  Interquartile range.

**Figure 3:**
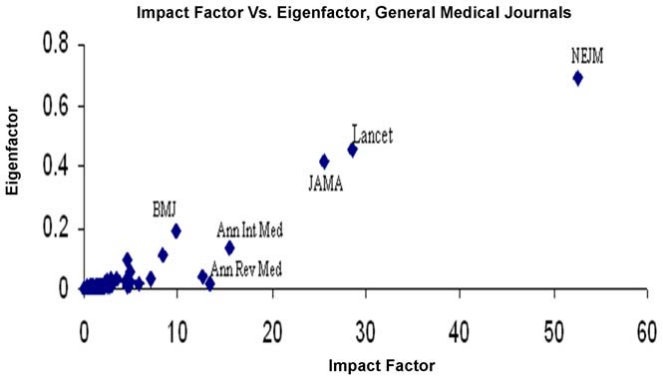
Impact Factor Versus Eigenfactor, General Medical Journals. IF,EF scores, 100 titles in ‘medicine, general and internal’, JCR 2007. Top journals, by IF  =  New England Journal of Medicine (NEJM), Lancet, Journal of American Medical Association (JAMA), Annals of Internal Medicine, & Annual Review of Medicine. Top 5, by EF  =  NEJM, Lancet, JAMA, British Medical Journal (BMJ) and Annals of Internal Medicine.

**Figure 4:**
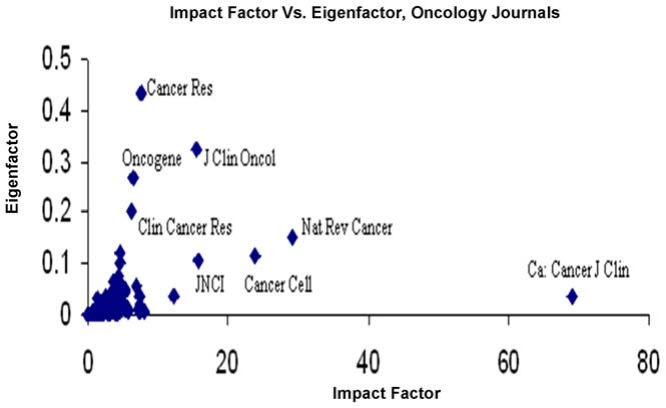
Impact Factor Versus Eigenfactor Scores, Oncology Journals. IF,EF scores for 132 titles in category ‘oncology’, JCR 2007. Top 5 journals, by IF  =  Ca:Cancer Journal Clinicians, Nature Reviews Cancer, Cancer Cell, the Journal of the National Cancer Institute, and the Journal of Clinical Oncology (JCO). Top 5, by EF  =  Cancer Research, the JCO, Oncogene, Clinical Cancer Research, and Nature Reviews Cancer.

As expected, there was a strong correlation between the EF and the IF in the general medical literature (r = 0.854, p = 0.000) (Figure 3), and this relationship was maintained in the top 20 journals by IF and EF (r = 0.674, p = 0.001). Strong correlation was also noted between EF and IF scores in the oncology literature (r = 0.725, p = 0.000) (Figure 4); this was not maintained in the highest impact journals (Groups BIF and BEF), however (r = 0.289, p = 0.217).

## Discussion

The relationship between a medical specialty or condition and its associated research output, with respect to volume and ‘quality’, is complex and may be dependent on such diverse influences as funding, socio-political influence and disease causation [18]. This complexity notwithstanding, the journal IF is increasingly used as a simple proxy measure of research productivity. Despite concerns that it was neither designed nor intended for this purpose, research funding is now frequently dependent upon it. Furthermore, the independence and objectivity of a commercially driven measure has been questioned. Until recently there was no viable alternative to the IF; the development of the EF at least offers stakeholders within the various scientific and medical disciplines an opportunity to assess research yield from a different perspective.

We have demonstrated that whilst oncology contributed about 8% of the output of medical journals in 2007, it represented 25% of output from the highest IF and EF journals. This disproportionate representation of oncology topics in more prestigious journals has been suggested before [19]; that these articles are dominated by a small number of cancers is a novel finding, however.

This level of bias to specific cancers is not limited to the general medical journals. Publications relating to prostate, breast, lung, and intestinal cancer, and leukaemia, accounted for over half of the total output in the top medical oncology journals. Analysis of the top 10 most prolific journals for each cancer reveals a similar picture; the journals publishing most frequently on these cancers are associated with much higher IF and EF scores.

It may be a source of concern for those working in less fashionable areas of oncology that a small number of cancers dominate the scientific yield. This domination notwithstanding however, our results support the argument that, relative to their impact on society, 4 of these cancers - breast, prostate, lung and intestinal - are actually underrepresented, with research interest in many of the rarer malignancies disproportionately greater. Furthermore, as much as 60% of cancer research is not ‘site specific’ and hence may hold relevance for all types of cancer [20]. In addition, whilst we focus on the relationship between disease burden and research output, there are many other influences on the level of research interest in a given area, including scientific opportunity; researchability; potential for progress; fundraising (certain tumours might attract more public donations than others); and the quality and size of the research workforce in different areas [20].

Our results have demonstrated that whilst there is significant correlation between the IF and EF in the oncology literature as a whole, differences exist both for the high impact oncology journals and at the level of individual cancers. If one measures article value on IF alone, then the highest-ranking oncology journal would be *CA: A Cancer Journal For Clinicians*. In contrast, *Cancer Research* is the highest-ranking oncology journal by EF (with *CA: A Cancer Journal for Clinicians* not making the top 20 oncology journals).

In addition, IF and EF do not correlate when assessing output on individual malignancies; for example, publications relating to testicular and renal cancers are published most frequently in high EF, but lower IF journals. In contrast, breast cancer, which has a high IF for the ten journals which published most frequently on it, is associated with a comparatively low EF score. Given that scientific impact is a multidimensional construct, the difference in IF and EF ranking may be an effect of relevance rather than quality of the article searched.

The top 5 oncology journals by EF in 2007 included at least one of the ten most prolific journals for 18 of the 26 cancers. The top 5 oncology journals by IF, however, published prolifically on just 11 of the 26 commonest cancers. What is the reason for this difference? The developers of the EF score described it as “the result of a random walk through the scientific literature. The algorithm corresponds to a basic model of research in which readers follow chains of citations as they move from journal to journal…..Because of the structure of the citation network, our model researcher will frequently visit large, important journals….and will seldom visit small journals in the lowest tiers of the publishing hierarchy” [8]. Our data demonstrates that those who choose to employ the EF as their discriminator will identify journals covering a greater breath of cancer topics than those identified using the IF, and suggests that the EF is not just an index of quality, but also functions as a measure of relevance, at least within oncology as a whole.

The above analysis notwithstanding, researchers need to be aware of the fact that literature regarding certain cancers may be limited to low ranking journals according to standard proxy measures. This may not be a reflection of the quality of the output so much as targeting of specific audiences [6]. This study has demonstrated the breadth of journal titles responsible for output in oncology and has identified which journals are most prolific in which sub-specialties. Even the most wide-ranging journals published on less than half of the 26 cancers included in this analysis; it is thus clear that, for many, a reliance on quality indicators, including the EF, in choosing which journals to search will result in poor retrieval of the most relevant information, and those involved in this process thus need to be cognisant of the situation within their particular sub-specialty.

This work has a number of limitations. Whilst bibliometric indicators can provide an interesting overview of scientific yield for a given subject area, they are nevertheless just proxy measures, and cannot replace the gold standard of reading each article and journal individually to assess their quality or otherwise. We examined English-language entries only; this obviously has implications for those cancers which have a greater disease burden in non-english speaking areas, wherein research output on those topics might be much greater and introduces a level of bias into our results. Furthermore, it should be noted that our study was limited to a single year and, whilst we tried to ensure that our searches were equivalent across the databases, it was not possible to ensure absolute uniformity in the search strategies used; hence the figures should be viewed in the context of the overall picture, rather than in absolute terms. In addition, our work does not concentrate specifically on research articles only, which some would argue to have been preferable, although we believe our search strategy gives a better indication of overall interest within each sub-discipline of oncology. Finally, our analysis of the 132 journals within the category ‘Oncology’ was based on the list provided by the 2007 JCR from Thomson Reuters; it should be noted that an alternative list could have been used based on the *SCImago Journal Rank* indicator (SJR) which is calculated within Scopus (Elsevier), but it has been previously demonstrated that those oncology journals indexed in Scopus which are not covered by the JCR tend to have low to very low impact factors.[21]

The controversy surrounding the use of bibliometric indicators notwithstanding, this analysis has demonstrated the privileged position which oncology holds in the medical literature. This preferential bias is not extended uniformly across the oncological spectrum and this heterogenicity requires closer scrutiny.

It is clear that the commonly used IF and the recently introduced EF do not correlate for the preeminent oncology journals, nor do they correlate at the level of individual cancers. Researchers should be aware that selection of one measure over the other as a proxy evaluation of quality may significantly change the strength of, for example, a grant proposal.

Finally, our results also suggest that the most relevant information for those working in many of the oncologic sub-specialties is not necessarily to be found in the most prestigious journals as delineated by proxy indicators of quality. This article raises significant questions regarding the best method of assessment of research and scientific output in the field of oncology.

## Supporting Information

Table S1 Incidence of Neoplasms, per 100,000 population, and Search Strategies Employed. Malignancies listed in decreasing order of incidence, as defined by the SEER Database, 2006. Rates are age-adjusted to the 2000 US standard population. TS  =  Topic subject.
(0.07 MB DOC)

Table S2 Top 20 Journals by Impact Factor and Eigenfactor. *Categories included in analysis of the top 20 medical journals  =  Gastroenterology and Hepatology; Haematology; Medicine, General & Internal; Medicine, Research & Experimental; Obstetrics and Gynaecology; Oncology; Orthopaedics; Otorhinolaryngology; Pathology; Paediatrics; Radiology, Nuclear Medicine & Medical Imaging; Respiratory System; Surgery; Transplantation; Urology & Nephrology.
(0.06 MB DOC)

Table S3 Top 10 Publishing Journals, per Malignancy, 2007. Where journals were responsible for an equivalent number of entries in tenth position, all are named. Calculations of median impact- and Eigenfactors were based on the ten journals with the highest ratings.
(0.19 MB DOC)
