# Supplementary material for: Representation of Cancer in the Medical Literature - A Bibliometric Analysis
Source: PLoS One. 2010 Nov 9;5(11):e13902. doi: 10.1371/journal.pone.0013902 (PMC2976696; doi:10.1371/journal.pone.0013902)
Supplement: Table S1 — Incidence of Neoplasms, per 100,000 population, and Search Strategies Employed. Malignancies listed in decreasing order of incidence, as defined by the SEER Database, 2006. Rates are age-adjusted to the 2000 US standard population. TS = Topic subject. (0.07 MB DOC) [file pone.0013902.s001.doc]

**Table S1**

**Incidence of Neoplasms, per 100,000 population, and Search Strategies Employed**

| **Neoplasm** | **Incidence** | **PubMed Search Term** | **Web of Science Search Terms** |
| --- | --- | --- | --- |
| Prostatic | 163.06 | Prostate neoplasms | TS=((prostate neoplasm$) OR (prostate cancer$) OR (prostate tumo$r$) OR (prostatic neoplasm$) OR (prostatic cancer$) OR (prostatic tumo$r$)) |
| Breast | 123.04 | Breast neoplasms | TS=((phyllodes tumo$r$) OR (Cystosarcoma Phyllo$des) OR (Malignant Cystosarcoma Phyllodes) OR (breast invasive ductal carcinoma) OR (infiltrating duct carcinoma$) OR (mammary ductal carcinoma$) OR (breast cancer) OR (breast neoplasm$) OR (breast tumo$r$) OR (human mammary neoplasm$) OR (human mammary carcinoma$)) |
| Lung | 59.97 | Lung neoplasms | TS=((lung neoplasm$) OR (lung cancer$) OR (lung tumo$r$) OR (pulmonary neoplasm$) OR (pulmonary cancer$) OR (pulmonary tumo$r$) OR (bronchogenic carcinoma$) OR (Bronchial Carcinoma$) OR (Non-Small-Cell Lung Carcinoma$) OR (Small Cell Lung Cancer) OR (Oat Cell Lung Cancer) OR (Oat Cell Carcinoma) OR (Small Cell Lung Carcinoma) OR (Multiple Pulmonary Nodule$) OR (Pancoast* Syndrome) OR (Pancoast Tumo$r) OR (Pulmonary Blastoma$) OR (Pulmonary Sclerosing Hemangioma$) OR (Lung Sclerosing Hemangioma$) OR (Solitary Pulmonary Nodule$) OR (Pulmonary Coin Lesion$)) |
| Intestinal | 48.88 | Intestinal neoplasms | TS=((intestinal neoplasm$) OR (intestinal cancer$) OR (Intestines Neoplasm$) OR (Intestines Cancer$) OR (Cecal Neoplasm$) OR (Cecal Cancer$) OR (Cecum Cancer$) OR (Appendiceal Neoplasm$) OR (Appendiceal Cancer$) OR (Appendix Cancer) OR (Colorectal Tumo$r$) OR (Colorectal Neoplasm$) OR (Colorectal Carcinoma$) OR (Colorectal Cancer$) OR (Familial Polyposis Syndrome) OR (Familial Polyposis Coli) OR (Familial Adenomatous Polyposis) OR (Familial Adenomatous Polyposis Coli) OR (Gardner Syndrome$) OR (Colonic Neoplasm$) OR (Colon Neoplasm$) OR (Colon Cancer$) OR (Colonic Cancer$) OR (Sigmoid Neoplasm$) OR (Sigmoid Colon Neoplasm$) OR (Sigmoid Cancer$) (Sigmoid Colon Cancer$) OR (Hereditary Nonpolyposis Colorectal Neoplasm$) OR (Hereditary Nonpolyposis Colorectal Cancer$) OR (Colon Cancer$ Familial Nonpolyposis) OR (Lynch Cancer$ Family Syndrome I) OR (Lynch Syndrome) OR (Rectal Neoplasm$) OR (Rectum Neoplasm$) OR (Rectal Tumo$r$) OR (Rectum Cancer$) OR (Rectal Cancer$) OR (Duodenal Neoplasm$) OR (Duodenal Cancer$) OR (Duodenum Cancer$) OR (Ileal Neoplasm$) OR (Ileal Cancer$) OR (ILEUM Cancer$) OR (Jejunal Neoplasm$) OR (Jejunal Cancer$) OR (Jejunum Cancer$) OR (Anal Neoplasm$) OR (Anal Cancer$) OR (Anus Neoplasm$) OR (Anus Cancer$) OR (Perianal Gland Neoplasm$) OR (Circumanal Gland Neoplasm$) OR (Anal Gland Neoplasm$)) |
| Melanoma | 21.14 | Melanoma | TS=((melanoma$) OR (malignant melanoma$) OR (Hutchinson* Melanotic Freckle) OR (Melanotic Freckle$) OR (Malignant Lentigo$) OR (Lentigo Maligna) OR (Amelanotic Melanoma$) OR (Experimental Melanoma$) OR (Cloudman S91 Melanoma) OR (Harding$Passey Melanoma) OR (B16 Melanoma$)) |
| Urinary | 20.46 | Urinary bladder neoplasms | TS=((Urinary Bladder Neoplasm$) OR (Bladder Tumo$r$) OR (Bladder Neoplasm$) OR (Urinary Bladder Cancer$) OR (Bladder Cancer$)) |
| Lymphoma | 19.52 | Lymphoma, Non-hodgkin | TS=((Non Hodgkin* Lymphoma$) OR (Nonhodgkin* Lymphoma$) OR (Diffuse Small Cleaved Cell Lymphoma$) OR (Lymphoma$ Atypical Diffuse Small Lymphoid) OR (Lymphoma$ High Grade) OR (Lymphoma$ Intermediate Grade) OR (Reticulum Cell Sarcoma$) OR (Reticulosarcoma$) OR (Lymphoma$ Mixed Cell) OR (Mixed Lymphocytic Histiocytic Lymphoma$) OR (Lymphoma$ Mixed) OR (Diffuse Mixed Cell Lymphoma$) OR (Diffuse Mixed Small and Large Cell Lymphoma$) OR (Small Non Cleaved Cell Lymphoma$) OR (Lymphoma$ Diffuse Mixed Lymphocytic Histiocytic) OR (Lymphoma$ Undifferentiated Diffuse) OR (Small Noncleaved Cell Lymphoma$) OR (Diffuse Undifferentiated Lymphoma$) OR (Undifferentiated Lymphoma$) OR (Pleomorphic Lymphoma$) OR (Diffuse Lymphoma$) OR (Lymphosarcoma$) OR (Lymphatic Sarcoma$) OR (Lymphoma$ Low Grade) OR (Burkitt* Lymphoma$) OR (Burkitt* Tumo$r$) OR (Burkitt Cell Leukemia$) OR (Burkitt* Leukemia$) OR (Leukemia$ Lymphoblastic Burkitt Type) OR (L3 Lymphocytic Leukemia$) OR (African Lymphoma$) OR (B Cell Lymphoma$) OR (AIDS Associated Lymphoma$) OR (Lymphom$ AIDS Related) OR (HIV Related Lymphoma$) OR (MALT Lymphoma$) OR (Lymphoma$ of Mucosa Associated Lymphoid Tissue) OR (Mucosa Associated Lymphoid Tissue Lymphoma$) OR (Marginal Zone B Cell Lymphoma$) OR (Diffuse Large Cell Lymphoma$) OR (Diffuse Large B Cell Lymphoma$) OR (Histiocytic Lymphoma$) OR (Diffuse Histiocytic Lymphoma$) OR (Large Lymphoid Lymphoma$ Diffuse) OR (Large Cell Lymphoma$) OR (Primary Effusion Lymphoma$)) TS=((Granulomatos$s Lymphomatoid) OR (Follicular Lymphoma$) OR (Nodular Lymphoma$) OR (Giant Follicular Lymphoma$) OR (Brill Symmers Disease) OR (Lymphoma$ Small Cleaved Cell Follicular) OR (Lymphoma$ Lymphocytic Nodular Poorly Differentiated) OR (Small Follicular Center Cell Lymphoma$) OR (Lymphoma$ Small Lymphoid Follicular) OR (Large Cell Lymphoma$ Follicular) OR (Lymphoma$ Histiocytic Nodular) OR (Nodular Large Follicular Center Cell Lymphoma$) OR (Large Lymphoid Lymphoma$ Nodular) OR (Lymphoma$ Follicular Grade 2) OR (Follicular Lymphoma$ Grade 3) OR (Lymphoma$ Follicular Mixed Cell) OR (Lymphoma$ Follicular Small and Large Cleaved Cell) OR (Lymphoma$ Follicular Mixed Lymphocytic Histiocytic) OR (Lymphoma$ Nodular Mixed Lymphocytic Histiocytic) OR (Lymphoma$ Nodular Mixed Small and Large Cell) OR (Lymphoma$ Follicular Grade 1) OR (Lymphoma$ Follicular Mixed Small and Large Lymphoid) OR (CD30 Positive Anaplastic Large Cell Lymphoma$) OR (Ki 1 Lymphoma$) OR (Systemic Anaplastic Large Cell Lymphoma$) OR (Anaplastic Large Cell Lymphoma$) OR (CD30+ Anaplastic Large Cell Lymphoma$) OR (Lymphoma Large Cell Ki 1) OR (Immunoblastic Sarcoma$) OR (Diffuse Immunoblastic Lymphosarcoma$) OR (Large Cell Immunoblastic Lymphoma$) OR (Immunoblastoma$) OR (Mantle Cell Lymphoma$) OR (Mantle Zone Lymphoma$) OR (Diffuse Lymphocytic Lymphoma$ Poorly Differentiated) OR (Centrocytic Small Cell Lymphoma$) OR (Lymphoma$ Lymphocytic Diffuse Intermediate Differentiated) OR (Lymphoma$ Lymphocytic Intermediate) OR (T Cell Lymphoma$)) |
| Uterine | 30.80 | Uterine neoplasms | TS=((Uterus Neoplasm$) OR (Uterine Neoplasm$) OR (Uterus Cancer$) OR (Uterine Cancer$) OR (Endometrial Neoplasm$) OR (Endometrial Carcinoma$) OR (Endometrium Cancer$) OR (Endometrium Carcinoma$) OR (Endometrial Cancer$) OR (Endometrioid Carcinoma$) OR (Endometrioid Adenocarcinoma$) OR (Endometrial Stromal Tumo$r$) OR (Sarcoma Endometrial Stromal Low-Grade) OR (Endolymphatic Stromal Myos*) OR (Endometrial Stromal Sarcoma$) OR (Uterine Cervical Neoplasm$) OR (Cervix Neoplasm$) OR (Cervical Neoplasm$) OR (Uterine Cervical Cancer$) OR (Cervix Cancer$)) |
| Kidney | 13.90 | Kidney neoplasms | TS=((kidney$ neoplasm$) OR (kidney$ cancer$) OR (kidney tumo$r*) OR (renal neoplasm$) OR (renal cancer$) OR (Renal Cell Adenocarcinoma$) OR (Renal Cell Carcinoma$) OR (Nephroid Carcinoma$) OR (Clear Cell Renal Cell Carcinoma$) OR (Hypernephroid Carcinoma$) OR (Hypernephroma$) OR (Clear Cell Renal Carcinoma$) OR (Grawitz Tumo$r$) OR (Papillary Renal Cell Carcinoma$) OR (Sarcomatoid Renal Cell Carcinoma$) OR (Chromophobe Renal Cell Carcinoma$) OR (Collecting Duct Carcinoma$ Kidney) OR (Mesoblastic Nephroma$) OR (Congenital Mesoblastic Nephroma$) OR (Wilm* Tumo$r$) OR (Nephroblastoma$) OR (Denys Drash Syndrome) OR (Wilms Tumor Pseudohermaphroditism) OR (WAGR Syndrome$) OR (WAGR Contiguous Gene Syndrome$) OR (WAGR Compl*) OR (Wilms Tumor Aniridia Genitourinary Anomalies MR Syndrome)) |
| Pancreatic | 12.01 | Pancreatic neoplasms | TS=((Pancreatic Neoplasm$) OR (Pancreas Neoplasm$) OR (Pancreas Cancer$) OR (Pancreatic Cancer$) OR (Islet Cell Adenoma$) OR (Islet Cell Tumo$r$) OR (Island Cell Tumo$r$) OR (Nesidioblastoma$) OR (Insulinoma$) OR (Insuloma$) OR (beta Cell Tumo$r$) OR (beta Cell Adenoma$) OR (Islet Cell Carcinoma$) OR (Islet Cell Tumo$r Malignant) OR (Gastrinoma$) OR (Ulcerogenic Islet Cell Tumo$r$) OR (Gastrin-Producing Tumo$r$) OR (Glucagonoma$) OR (alpha Cell Tumo$r$) OR (alpha-Cell Adenoma$) OR (Somatostatinoma$) OR (Vipoma$) OR (VIP Secreting Tumo$r$) OR (Diarrheogenic Islet Cell Tumo$r$) OR (Diarrheogenic Tumo$r$) OR (Pancreatic Cholera) OR (Watery Diarrhea Syndrome) OR (Vipoma Syndrome) OR (WDHA) OR (WDHH) OR (Verner Morrison Syndrome) OR (Watery Diarrhea with Hypokalemic Alkalosis) OR (Pancreatic Ductal Carcinoma$) OR (Duct-Cell Carcinoma$ Pancreas) OR (Pancreatic Duct Cell Carcinoma$) OR (Pancreatic Ductal Carcinoma$) OR (Ductal Carcinoma of the Pancreas)) |
| Leukemia | 11.89 | Leukaemia | TS=(leukemia$) |
| Thyroid | 11.03 | Thyroid neoplasms | TS=((Thyroid Neoplasm$) OR (Thyroid Carcinoma$) OR (Thyroid Cancer$) OR (Thyroid Adenoma$) OR (Thyroid Nodule$)) |
| Mouth | 10.29 | Mouth neoplasms | TS=((Mouth Neoplasm$) OR (Oral Neoplasm$) OR (Mouth Cancer$) OR (Oral Cancer$) OR (Gingival Neoplasm$) OR (Congenital Epuli*) OR (Oral Leukoplakia$) OR (Oral Leukokeratos*) OR (Keratosis Oral) OR (Hairy Leukoplakia$) OR (Oral Hairy Leukoplakia$) OR (Lip Neoplasm$) OR (Lip Cancer$) OR (Palatal Neoplasm$) OR (Salivary Gland Neoplasm$) OR (Salivary Gland Cancer$) OR (Parotid Neoplasm$) OR (Parotid Cancer$) OR (Sublingual Gland Neoplasm$) OR (Submandibular Gland Neoplasm$) OR (Tongue Neoplasm$) OR (Tongue Cancer$)) |
| Stomach | 7.34 | Stomach neoplasms | TS=((Stomach Neoplasm$) OR (Gastric Neoplasm$) OR (Stomach Cancer$) OR (Gastric Cancer$)) |
| Liver | 6.69 | Liver neoplasms | TS=((Liver Neoplasm$) OR (Hepatic Neoplasm$) OR (Hepatic Cancer$) OR (Liver Cancer$) OR (Liver Cell Adenoma$) OR (Benign Hepatoma$) OR (Hepatocellular Adenoma$) OR (Hepatocellular Carcinoma$) OR (Hepatoma$) OR (Experimental Liver Neoplasm$) OR (Morris Hepatoma) OR (Novikoff Hepatoma) OR (Experimental Hepatoma$)) |
| Ovarian | 12.48 | Ovarian neoplasms | TS=((Ovarian Neoplasm$) OR (Ovary Neoplasm$) OR (Ovary Cancer$) OR (Ovarian Cancer$) OR (Granulosa Cell Tumo$r$) OR (Granulosa Cell$ Cancer$) OR (Luteoma$) OR (Luteinoma$) OR (Pregnancy Luteoma$) OR (Gestational Luteoma$) OR (Meig* Syndrome) OR (Sertoli Leydig Cell Tumo$r$) OR (Androblastoma$) OR (Arrhenoblastoma$) OR (Tumo$r$ Leydig Cell) OR (Interstitial Cell Tumo$r$) OR (Tumo$r$ Sertoli Cell) OR (Thecoma$) OR (Theca Cell Tumo$r$)) |
| CNS | 6.12 | Central nervous system neoplasms | TS= ((Neoplasm$ Central Nervous System) OR (Central Nervous System Tumo$r$) OR (Brain Neoplasm$) OR (Brain Tumo$r$) OR (Brain Cancer$) OR (Intracranial Neoplasm$) OR (Cerebroventricular Neoplasm$) OR (Intraventricular Neoplasm$) OR (Cerebral Ventricle Tumo$r$) OR (Brain Ventricular Tumo$r$) OR (Brain Ventricular Neoplasm$) OR (Choroid Plexus Tumo$r$) OR (Choroid Plexus Neoplasm$) OR (Choroid Plexus Papilloma$) OR (Posterior Fossa Tumo$r$) OR (Posterior Fossa Neoplasm$) OR (Infratentorial Neoplasm$) OR (Infratentorial Tumo$r$) OR (Infratentorial Cancer$) OR (Brain Stem Neoplasm$) OR (Brainstem Tumo$r$) OR (Brainstem Neoplasm$) OR (Brain Stem Tumo$r$) OR (Pontine Tumo$r$) OR (Pontine Neoplasm$) OR (Mesencephalic Neoplasm$) OR (Midbrain Neoplasm$) OR (Midbrain Tumo$r$) OR (Medullary Neoplasm$) OR (Medullary Tumo$r$) OR (Cerebellar Tumo$r$) OR (Cerebellar Neoplasm$) OR (Cerebellum Primary Neoplasm$) OR (Cerebellar Cancer$) OR (Neurocytoma$) OR (Pinealoma$) OR (Pineal Tumo$r$) OR (Pineal Neoplasm$) OR (Pineoblastoma$) OR (Mixed Pineocytoma Pineoblastoma$) OR (Pinealocytoma$) OR (Pineocytoma$) OR (Supratentorial Tumo$r$) OR (Supratentorial Neoplasm$) OR (Supratentorial Cancer$)) OR ((Hypothalam* Neoplasm$) OR (Hypothalam* Tumo$r$) OR (Hypothalamic Cancer$) OR (Hypothalamic Pituitary Neoplasm$) OR (Hypothalamo Neurohypophysial Region Neoplasm$) OR (Hypothalamic Teratoma$) OR (Hypothalamic Chiasmatic Neoplasm$) OR (Benign Hypothalamic Neoplasm$) OR (Pallister Hall Syndrome) OR (Pituitary Neoplasm$) OR (Pituitary Tumo$r$) OR (Pituitary Adenoma$) OR (Pituitary Carcinoma$) OR (Pituitary Cancer$) OR (Nelson Syndrome) OR (Somatotroph Adenoma$) OR (Corticotroph Adenoma$) OR (Prolactinoma$) OR (Lactotroph Adenoma$) OR (M$croprolactinoma$) OR (Cyst$ Central Nervous System) OR (Rathke* Cleft Cyst$) OR (Suprasellar Cyst$) OR (Arachnoid Cyst$) OR (Arachnoid Diverticula$) OR (Leptomeningeal Cyst$) OR (Intracranial Arachnoid Cyst$) OR (Meningeal Neoplasm$) OR (Meningeal Tumo$r$) OR (Leptomeningeal Neoplasm$) OR (Meningeal Cancer$) OR (Benign Meningeal Neoplasm$) OR (Meningeal Carcinomatos$s) OR (Carcinomatous Meningiti*) OR (Leptomeningeal Carcinomatos$s) OR (Meningioma$) OR (Meningiomatos$s) OR (Spinal Cord Tumo$r$) OR (Spinal Cord Neoplasm$) OR (Epidural Neoplasm$) OR (Epidural Tumo$r$)) |
| Myeloma | 5.37 | Multiple myeloma | TS=((multiple myeloma$) OR (Myeloma$ Plasma Cell) OR (Plasma Cell Leukemia$) OR (Plasmacytic Leukemia$)) |
| Esophageal | 4.56 | Esophageal neoplasms | TS=((esophageal neoplasm$) OR (esophageal cancer$) OR (esophagus neoplasm$) OR (esophagus cancer$)) |
| Laryngeal | 3.24 | Laryngeal neoplasms | TS=((Laryngeal Neoplasm$) OR (Larynx Neoplasm$) OR (Larynx Cancer$) OR (Laryngeal Cancer$)) |
| Soft Tissue | 2.98 | Soft tissue neoplasms | TS=((soft tissue neoplasm$) OR (muscle neoplasm$) OR (muscle cancer$) OR (vascular neoplasm$) OR (heart neoplasm$) OR (cardiac tumo$r$) OR (cardiac neoplasm$) OR (cardiac carcinoma$) OR (heart cancer$) OR (cardiac cancer$)) |
| Hodgkin | 2.93 | Hodgkin disease | TS=((hodgkin* disease$) OR (Hodgkin* Lymphoma$) OR (Malignant Lymphogranuloma$) OR (Hodgkin* Lymphoma) OR (Hodgkin* Granuloma) OR (Malignant Granuloma$)) |
| Testicular | 5.52 | Testicular neoplasms | TS=((Testis Neoplasm$) OR (Testicular Neoplasm$) OR (Testis Cancer$) OR (Testicular Cancer$) OR (Rete Testis Tumo$r$) OR (Sertoli Leydig Cell Tumo$r$) OR (Androblastoma$) OR (Arrhenoblastoma$) OR (Tumo$r$ Leydig Cell) OR (Interstitial Cell Tumo$r$) OR (Tumo$r$ Sertoli Cell)) |
| Vulvar | 2.32 | Vulvar neoplasms | TS=((Vulva$ Neoplasm$) OR (Vulva$ Cancer$)) |
| Gallbladder | 1.11 | Gallbladder neoplasms | TS=((gallbladder neoplasm$) OR (gallbladder cancer$) OR (gall bladder neoplasm$) OR (gall bladder cancer$)) |
| Mesothelioma | 0.93 | Mesothelioma | TS=((mesothelioma$)) |

Malignancies listed in decreasing order of incidence, as defined by the SEER Database, 2006. Rates are age-adjusted to the 2000 US standard population. TS = Topic subject
