# Supplementary material for: Representation of Cancer in the Medical Literature - A Bibliometric Analysis
Source: PLoS One. 2010 Nov 9;5(11):e13902. doi: 10.1371/journal.pone.0013902 (PMC2976696; doi:10.1371/journal.pone.0013902)
Supplement: Table S2 — Top 20 Journals by Impact Factor and Eigenfactor. *Categories included in analysis of the top 20 medical journals = Gastroenterology and Hepatology; Haematology; Medicine, General & Internal; Medicine, Research & Experimental; Obstetrics and Gynaecology; Oncology; Orthopaedics; Otorhinolaryngology; Pathology; Paediatrics; Radiology, Nuclear Medicine & Medical Imaging; Respiratory System; Surgery; Transplantation; Urology & Nephrology. (0.06 MB DOC) [file pone.0013902.s002.doc]

**Table S2**

Top 20 Journals By Impact Factor and Eigenfactor

| **AIF*** | **IF** | **AEF*** | **EF** |
| --- | --- | --- | --- |
| CA-CANCER J CLIN | 69.026 | NEW ENGL J MED | 0.69405 |
| NEW ENGL J MED | 52.589 | CIRCULATION | 0.53421 |
| NAT REV CANCER | 29.19 | BLOOD | 0.46953 |
| LANCET | 28.638 | LANCET | 0.45171 |
| NAT MED | 26.382 | CANCER RES | 0.43681 |
| JAMA | 25.547 | JAMA | 0.41748 |
| CANCER CELL | 23.858 | J CLIN ONCOL | 0.32292 |
| J CLIN INVEST | 16.915 | J EXP MED | 0.28631 |
| J NATL CANCER I | 15.678 | J CLIN INVEST | 0.28017 |
| J EXP MED | 15.612 | ONCOGENE | 0.26873 |
| ANN INTERN MED | 15.516 | NAT MED | 0.23469 |
| J CLIN ONCOL | 15.484 | CLIN CANCER RES | 0.20223 |
| ANNU REV MED | 13.415 | BRIT MED J | 0.18931 |
| CIRCULATION | 12.755 | GASTROENTEROLOGY | 0.15861 |
| PLOS MED | 12.601 | NEUROIMAGE | 0.15479 |
| LANCET ONCOL | 12.247 | NAT REV CANCER | 0.15048 |
| GASTROENTEROLOGY | 11.673 | CIRC RES | 0.1498 |
| BLOOD | 10.896 | ANN INTERN MED | 0.13553 |
| HEPATOLOGY | 10.734 | PEDIATRICS | 0.13523 |
| GUT | 10.015 | AM J RESP CRIT CARE | 0.12449 |
| **BIF** | **IF** | **BEF** | **EF** |
| CA-CANCER J CLIN | 69.026 | CANCER RES | 0.43681 |
| NAT REV CANCER | 29.19 | J CLIN ONCOL | 0.32292 |
| CANCER CELL | 23.858 | ONCOGENE | 0.26873 |
| J NATL CANCER I | 15.678 | CLIN CANCER RES | 0.20223 |
| J CLIN ONCOL | 15.484 | NAT REV CANCER | 0.15048 |
| LANCET ONCOL | 12.247 | CANCER | 0.12063 |
| NAT CLIN PRACT ONCOL | 8.217 | INT J CANCER | 0.11998 |
| CANCER RES | 7.672 | CANCER CELL | 0.11776 |
| STEM CELLS | 7.531 | J NATL CANCER I | 0.10638 |
| ADV CANCER RES | 7.524 | BRIT J CANCER | 0.10172 |
| SEMIN CANCER BIOL | 7.51 | INT J RADIAT ONCOL | 0.07661 |
| BBA-REV CANCER | 7.264 | EXP CELL RES | 0.0651 |
| LEUKEMIA | 6.924 | CANCER EPIDEM BIOM | 0.06306 |
| ONCOGENE | 6.44 | LEUKEMIA | 0.0562 |
| CLIN CANCER RES | 6.25 | ANN ONCOL | 0.05134 |
| NEURO-ONCOLOGY | 5.806 | EUR J CANCER | 0.0513 |
| NEOPLASIA | 5.674 | CARCINOGENESIS | 0.05116 |
| J PATHOL | 5.423 | CANCER LETT | 0.04524 |
| CARCINOGENESIS | 5.406 | J PATHOL | 0.04307 |
| CURR CAN DRUG TAR | 5.385 | MOL CANCER THER | 0.04051 |

Legend: *Categories included in analysis of the top 20 medical journals = Gastroenterology and Hepatology; Haematology; Medicine, General & Internal; Medicine, Research & Experimental; Obstetrics and Gynaecology; Oncology; Orthopaedics; Otorhinolaryngology; Pathology; Paediatrics; Radiology, Nuclear Medicine & Medical Imaging; Respiratory System; Surgery; Transplantation; Urology & Nephrology.
