# Supplementary material for: Representation of Cancer in the Medical Literature - A Bibliometric Analysis
Source: PLoS One. 2010 Nov 9;5(11):e13902. doi: 10.1371/journal.pone.0013902 (PMC2976696; doi:10.1371/journal.pone.0013902)
Supplement: Table S3 — Top 10 Publishing Journals, per Malignancy, 2007. Where journals were responsible for an equivalent number of entries in tenth position, all are named. Calculations of median impact- and Eigenfactors were based on the ten journals with the highest ratings. (0.19 MB DOC) [file pone.0013902.s003.doc]

**Table S3,**

Top 10 Publishing Journals, Per Malignancy, 2007

| **Prostate** | **N** | **Breast** | **N** | **Lung** | **N** | **Intestinal** | **N** |
| --- | --- | --- | --- | --- | --- | --- | --- |
| Eur Urol | 253 | Breast Cancer Res Treat | 554 | Lung Cancer | 332 | Int J Colorectal Dis | 165 |
| BJU Int | 230 | J Clin Oncol | 205 | J Thorac Oncol | 206 | World J Gastroenterol | 132 |
| J Urol | 195 | Cancer Res | 199 | Cancer Res | 128 | Dis Colon Rectum | 128 |
| Prostate | 161 | Breast | 168 | J Clin Oncol | 100 | Cancer Res | 127 |
| Urology | 155 | Ann Oncol | 143 | Int J Radiat Oncol Biol Phys | 99 | Ann Surg Oncol | 122 |
| Int J Radiat Oncol Biol Phys | 147 | Ann Surg Oncol | 143 | Ann Thorac Surg | 88 | Colorectal Dis | 116 |
| Cancer Res | 142 | Breast J | 137 | Chest | 88 | Int J Cancer | 98 |
| Prostate Cancer Pros Dis | 87 | Breast Cancer Res | 121 | Clin Cancer Res | 88 | J Clin Oncol | 93 |
| Cancer | 68 | Int J Cancer | 112 | Cancer | 74 | Endoscopy | 82 |
| Clin Cancer Res | 67 | Br J Cancer | 108 | Eur J Cardiothorac Surg | 70 | Br J Cancer | 81 |
|  |  |  |  |  |  |  |  |
| **Melanoma** | **N** | **Urinary** | **N** | **Lymphoma (Non-hodgkin)** | **N** | **Uterine** | **N** |
| Cancer Res | 68 | Eur Urol | 100 | Leuk Lymphoma | 153 | Gynecol Oncol | 337 |
| Melanoma Res | 59 | J Urol | 89 | Blood | 117 | Int J Gynecol Cancer | 173 |
| J Invest Dermatol | 47 | Urology | 62 | Br J Haematol | 68 | Am J Obstet Gynecol | 55 |
| Br J Dermatol | 45 | BJU Int | 55 | Leukemia | 66 | Eur J Gynaecol Oncol | 53 |
| Clin Cancer Res | 43 | Urol Oncol | 35 | Haematologica | 53 | Int J Cancer | 50 |
| Cancer Immunol Immunother | 38 | Int J Urol | 27 | Ann Oncol | 52 | Int J Radiat Oncol Biol Phys | 48 |
| Int J Cancer | 36 | Int Urol Nephrol | 18 | J Clin Oncol | 49 | Cancer | 47 |
| Arch Dermatol | 35 | World J Urol | 18 | Ann Hematol | 40 | Eur J Obstet Gynecol Reprod Biol | 46 |
| J Cutan Pathol | 35 | Cancer | 17 | Eur J Haematol | 39 | Fertil Steril | 46 |
| Oncogene, Ann Surg Oncol, J Am Acad Dermatol | 31 | Int J Cancer | 17 | Leuk Res | 39 | Int J Gynaecol Obstet | 40 |
|  |  |  |  |  |  |  |  |
| **Kidney** | **N** | **Pancreatic** | **N** | **Leukaemia** | **N** | **Thyroid** | **N** |
| Eur Urol | 98 | Pancreas | 72 | Blood | 357 | Thyroid | 84 |
| J Urol | 92 | J Gastrointest Surg | 60 | Leukemia | 352 | J Clin Endocrinol Metab | 50 |
| Urology | 79 | Hepatogastroenterology | 52 | Leuk Res | 294 | World J Surg | 33 |
| BJU Int | 66 | Cancer Res | 51 | Leuk Lymphoma | 167 | Clin Nucl Med | 30 |
| Clin Cancer Res | 51 | JOP | 46 | Br J Haematol | 161 | Surgery | 23 |
| Int J Urol | 31 | World J Gastroenterol | 36 | Haematologica | 101 | Clin Endocrinol (Oxf) | 19 |
| J Endourol | 31 | Gastrointest Endosc | 31 | Bone Marrow Transplant | 89 | Eur J Endocrinol | 18 |
| Int Urol Nephrol | 25 | Ann Surg Oncol | 29 | Cancer Genet Cytogenet | 89 | Eur J Nucl Med Mol Imaging | 17 |
| Ann Oncol | 24 | Clin Cancer Res | 27 | Pediatr Blood Cancer | 77 | Endocr Relat Cancer | 16 |
| J Clin Oncol | 20 | Int J Cancer | 27 | J Clin Oncol | 70 | Ann Surg Oncol, Arq Bras Endocrinol Metabol | 16 |
|  |  |  |  |  |  |  |  |
| **Mouth** | **N** | **Stomach** | **N** | **Liver** | **N** | **Ovarian** | **N** |
| Oral Oncol | 144 | World J Gastroenterol | 76 | World J Gastroenterol | 128 | Gynecol Oncol | 244 |
| Oral Surg Oral Med Oral Pathol Oral Radiol Endod | 58 | Hepatogastroenterology | 67 | Hepatogastroenterology | 109 | Int J Gynecol Cancer | 137 |
| J Oral Maxillofac Surg | 51 | Ann Surg Oncol | 61 | Eur J Surg Oncol | 76 | J Clin Oncol | 60 |
| Br J Oral Maxillofac Surg | 46 | Dig Dis Sci | 43 | Ann Surg Oncol | 73 | Cancer Res | 57 |
| Int J Oral Maxillofac Surg | 43 | Gastric Cancer | 40 | J Gastroenterol Hepatol | 61 | Clin Cancer Res | 52 |
| J Oral Pathol Med | 43 | J Gastroenterol Hepatol | 35 | Hepatology | 59 | Eur J Gynaecol Oncol | 38 |
| Anticancer Res | 31 | Br J Cancer | 34 | J Gastrointest Surg | 47 | Br J Cancer | 31 |
| Eur Arch Otorhinolaryngol | 27 | Endoscopy | 29 | J Hepatol | 47 | Int J Gynecol Pathol | 31 |
| Oncol Rep | 26 | Cancer Sci | 26 | Liver Int | 46 | Ann Oncol | 29 |
| Otolaryngol Head Neck Surg | 26 | Int J Cancer | 26 | Cancer Res, Dig Dis Sci | 44 | Arch Gynecol Obstet | 29 |
|  |  |  |  |  |  |  |  |
| **CNS** | **N** | **Myeloma** | **N** | **Esophageal** | **N** | **Laryngeal** | **N** |
| J Neurooncol | 255 | Blood | 81 | Dis Esophagus | 45 | Eur Arch Otorhinolaryngol | 36 |
| J Neurosurg | 128 | Br J Haematol | 61 | Endoscopy | 41 | J Laryngol Otol | 28 |
| Neurosurgery | 96 | Leukemia | 53 | Ann Surg Oncol | 33 | Auris Nasus Larynx | 26 |
| Childs Nerv Syst | 91 | Leuk Lymphoma | 37 | Gastrointest Endosc | 29 | Otolaryngol Head Neck Surg | 21 |
| Int J Radiat Oncol Biol Phys | 79 | Haematologica | 31 | Ann Thorac Surg | 24 | Laryngoscope | 19 |
| Surg Neurol | 72 | Bone Marrow Transplant | 29 | Am J Gastroenterol | 23 | Head Neck | 15 |
| J Clin Neurosci | 67 | Leuk Res | 26 | World J Gastroenterol | 20 | Int J Radiat Oncol Biol Phys | 15 |
| Acta Neurochir (Wien) | 65 | Eur J Haematol | 25 | Anticancer Res | 19 | Arch Otolaryngol Head Neck Surg | 13 |
| AJNR Am J Neuroradiol | 50 | Ann Hematol | 23 | Oncol Rep | 19 | Acta Otolaryngol | 12 |
| J Clin Oncol, Clin Cancer Res | 49 | Clin Cancer Res | 22 | J Clin Oncol, Surg Endosc  Int J Radiat Oncol Biol Phys | 18 | Ann Otol Rhinol Laryngol,  Ear Nose Throat J, J Voice | 9 |
|  |  |  |  |  |  |  |  |
| **Soft Tissue & Heart** | **N** | **Hodgkin Lymphoma** | **N** | **Testicular** | **N** | **Vulvar** | **N** |
| Int J Cardiol | 38 | Leuk Lymphoma | 30 | Int J Androl | 27 | Gynecol Oncol | 24 |
| Skeletal Radiol | 25 | Blood | 28 | J Urol | 24 | Int J Gynecol Cancer | 14 |
| Cardiovasc Pathol | 20 | J Clin Oncol | 25 | Eur Urol | 22 | Eur J Gynaecol Oncol | 9 |
| J Thorac Cardiovasc Surg | 19 | Ann Oncol | 14 | J Clin Oncol | 22 | J Low Genit Tract Dis | 7 |
| J Am Soc Echocardiogr | 18 | Hematol Oncol Clin North Am | 14 | Urology | 21 | J Reprod Med | 7 |
| Ann Surg Oncol | 17 | Br J Haematol | 13 | Urol Clin North Am | 17 | Int J Gynecol Pathol | 6 |
| Cancer | 14 | Haematologica | 13 | BJU Int | 12 | J Clin Pathol | 6 |
| Am J Surg Pathol | 13 | Int J Cancer | 10 | Int J Urol | 11 | Acta Obstet Gynecol Scand | 4 |
| Ann Thorac Surg | 13 | Cancer | 8 | Ann Oncol | 10 | Int J Gynaecol Obstet | 4 |
| Clin Orthop Relat Res | 13 | Bone Marrow Transplant | 7 | N Engl J Med | 10 | Eur J Obstet Gynecol Reprod Biol | 3 |
|  |  |  |  |  |  |  |  |
| **Gallbladder (1-5)** | **N** | **Gallbladder (6-10)** | **N** | **Mesothelioma (1-5)** | **N** | **Mesothelioma (6-10)** | **N** |
| J Hepatobiliary Pancreat Surg | 12 | World J Gastroenterol | 5 | Lung Cancer | 16 | Ann Surg Oncol | 7 |
| J Gastrointest Surg | 10 | Hepatogastroenterology | 4 | J Thorac Oncol | 12 | Eur J Cardiothorac Surg | 7 |
| Ann Surg Oncol | 6 | Am J Surg  J Gastroenterol, Dig Dis Sci, Ann R Coll Surg Eng, Jpn J Clin Onocol, Ann Diag Pathol | 3 | Regul Toxicol Pharmacol | 11 | Int J Radiat Oncol BiolPhys | 7 |
| J Surg Oncol | 6 |  |  | Clin Cancer Res | 8 | Thorax, Cancer, Am J Clin Pathol, Int J Occ Environ Health,Occup Environ Medicine | 6 |
| J Gastroenterol Hepatol | 5 |  |  | Diagn Cytopathol | 8 |  |  |
|  |  |  |  |  |  |  |  |

Where journals were responsible for an equivalent number of entries in tenth position, all are named. Calculations of median impact- and Eigenfactors were based on the ten journals with the highest ratings.
